# The impact of perceived teacher support on students’ learning approach: the chain mediating role of academic engagement and achievement goal orientation

**DOI:** 10.3389/fpsyg.2025.1513538

**Published:** 2025-06-18

**Authors:** Yaqi Zhang, Xiangli Guan, Jingjing Wang, Sumei Yin, Xuejiao Li, Yang Li, Mary C. Jobe, Md Zahir Ahmed

**Affiliations:** ^1^School of Teacher Education, Yuxi Normal University, Yuxi, China; ^2^Department of Psychological and Brain Sciences, The George Washington University, Washington, DC, United States; ^3^School of Psychology, Zhejiang Normal University, Jinhua, China

**Keywords:** perceived teacher support, learning approach, academic engagement, achievement goal orientation, mastery-avoidance goals, performance-approach goals

## Abstract

**Introduction:**

This present study investigated the influential effects of university students’ perceived teacher support on their learning approach by drawing on Personal Investment Theory and selecting academic engagement, mastery-avoidance goals and performance-approach goals as mediating variables. A conceptual model between these variables was constructed based on existing research and Personal Investment Theory.

**Methods:**

Self-report data from 543 Chinese university undergraduates was analyzed through whole group sampling to verify the model and clarify the underlying mechanisms by which perceived teacher support influences university students’ learning approach. The study also examined the chain mediatory effects of academic engagement, mastery-avoidance goals, and performance-approach goals.

**Results:**

The findings suggest that perceived teacher support directly and positively predicts students’ learning approach, while perceived teacher support indirectly predicts students’ learning approach through a chain of academic engagement, mastery-avoidance goals and performance-approach goals. Additionally, this study explores the mechanisms by which extrinsic and intrinsic factors influence the learning approach of university students and presents the process of forming the learning approach of university students.

**Discussion:**

The results of this study emphasize the significance and importance of improving the literacy of university teachers in terms of their attention to the academic engagement and achievement motivation of university students.

## 1 Introduction

Students’ learning approaches significantly impact their academic performance, educational attainment, and instructors’ effectiveness (Keerthigha and Singh, 2023). Understanding learning approaches enhances student outcomes and helps educators design effective teaching strategies. Specifically, studying learning approaches facilitates personalized education by offering individualized programs and resources (Lockspeiser and Kaul, 2016). A deeper understanding maximizes students’ potential and improves both learning experiences and education quality. Identifying the factors influencing learning approaches, along with their interactions, provides a theoretical foundation for developing improved strategies. Among the various factors affecting learning approaches, teacher-related influences are particularly significant (Zheng, 2022; Cents-Boonstra et al., 2020; Howe et al., 2019). However, knowledge gaps remain regarding how teacher interactions shape different learning approaches, such as deep and surface learning. This study aims to fill the knowledge gap in the existing literature on the complex mediating mechanisms through which teacher support influences students’ learning styles. Although existing studies have confirmed the direct effects of teacher support on learning motivation and academic performance, there is still a lack of systematic exploration of the dynamic interactions of mediating pathways. Specifically, existing studies have only separately examined the single mediating effect of learning engagement or achievement goal orientation, failing to reveal how the two form a chain mediating mechanism, i.e., whether teacher support reinforces students’ achievement goal orientation by facilitating their learning engagement, which then influences the choice of students’ learning strategies. In addition, the mechanism of the differentiated role of different types of goal orientations, especially mastery avoidance goals and performance convergence goals, in this chain mediation pathway has not been clarified, and in particular, whether mastery avoidance goals and performance convergence goals have a moderating effect when they act as a secondary mediator is still theoretically controversial. The innovation of this study is to construct and validate the chain mediation model of “support-input-goal-oriented-learning style,” to reveal the dynamics of multidimensional psychological variables in educational and teaching contexts, and to provide new theoretical perspectives for a deeper understanding of the boundaries of the role of teacher support and the mechanism of hierarchical influence. This is especially relevant in light of recent reforms emphasizing personalized, student-centered strategies.

This present study explores the role of perceived teacher support for deep or surface level learning of students, with the goal of enhancing teaching strategies in diverse learning settings. Integrating the teacher-student interaction from educational psychology perspectives, it endeavors to extend the theoretical framework of learning approaches and offers practical implications at the policy and teaching level. Often, the students’ perception of teacher support is utilized as an indication of teacher influence. Perceived teacher support specifically signifies the students’ perceptions of teachers’ attitudes and behaviors within the learning context (Sadoughi and Hejazi, 2022; Babad, 1990). However, an isolated examination of perceived teacher support does not fully capture the underlying influential mechanism of learning approach. Previous studies have demonstrated that academic engagement and achievement goal orientation have moderating effects on perceived teacher support, emphasizing the emotional and motivational dimensions (Liu et al., 2021). These mediators underscore the complexity of teacher-student interactions and highlight the need to explore how academic engagement and achievement goal orientation influence learning approaches (Fredricks et al., 2004).

The self-regulation of learning approaches, involving academic engagement and achievement goal orientation, is explained by Personal Investment Theory, which posits that motivation results from how individuals invest time and resources based on personal incentives, self-beliefs, and perceived options (Zhang, 2023; Duda et al., 1989). Personal incentives are the internal subjectivities that drive and motivate students to engage in learning activities. Additionally, teacher support complements these incentives, subsequently ensuring meaningful learning engagement for students. In this context, self-beliefs pertain to students’ sense of competence, shaped by the teacher’s encouragement and constructive feedback (Barni et al., 2019; Ayllón et al., 2019). Such encouragement from teachers enhances students’ self-efficacy and motivates them to engage in complex learning goals. Finally, perceived options reflect how aware students are of adopting the most suitable strategy from the available alternatives. Ideally, teachers offer diversified methods to widen students’ strategic adaptation aligned with their learning goals (Duda et al., 1989). Incorporating perceived teacher support within Personal Investment Theory highlights how such support influences learning approaches by building students’ self-beliefs and broadening their perceived options in line with their goals. This study offers insights into how teacher support affects student motivation and engagement, potentially suggest improving teaching quality through personalized strategies.

### 1.1 The association of perceived teacher support with student learning approach

Perceived teacher support encompasses the emotional, informational, and appraisal support educators provide to students, fostering a supportive learning environment that directly influences student engagement and motivation (Sadoughi and Hejazi, 2022). Learning approaches involve not only what and how students learn but also their autonomy and responsibility in the learning process (Kember et al., 2004). Self-directed learning reflects higher levels of activity, with deep learning approaches considered ideal for improving learning outcomes, as opposed to surface approaches, which are associated with superficial understanding (Kember et al., 2004; Bryson and Hand, 2007). High perceived teacher support encourages students to adopt deep learning approaches, focusing on thoroughly understanding content and integrating it with prior knowledge (Baeten et al., 2010; Kikas et al., 2016). Effective teaching strategies—such as inquiry-based methods, technology-enhanced tools, and cooperative learning and problem-based learning—foster student-material interaction and critical reflection (Kalina and Powell, 2009). Emotional and social support from teachers plays a crucial role in promoting intrinsic motivation and engagement (Sezgin Selçuk et al., 2009). Additionally, teachers who guide students in developing metacognitive strategies enhance both self-efficacy and academic engagement (Sezgin Selçuk et al., 2009; Taghani and Razavi, 2021). While perceived teacher support positively influences learning approaches, complex interactions exist. Individual differences among students moderate the relationship, impacting how PTS shapes their learning (Klem and Connell, 2004). Research suggests that teachers adjust their support based on students’ needs and learning styles, which influences the effectiveness of PTS (Haar et al., 2002). However, inconsistent findings highlight that the relationship between perceived teacher support and learning approach varies depending on the teaching style—positive in teacher-dominated classrooms but negative in student-centered environments (Kikas et al., 2016). Perceived teacher support influences students’ learning approach by mitigating adverse learning experiences, such as anxiety and burnout, and one study found that teachers with high levels of support were more inclined to use inclusive teaching strategies, which in turn led to increased student learning satisfaction and learning resilience mentioned (Niluminda et al., 2025). These variations suggest the need to explore third-variable factors that influence the perceived teacher support -learning approach relationship.

### 1.2 The mediating role of academic engagement

Perceived teacher support does not always directly influence students’ learning approach, as mediating variables such as academic engagement play a significant role (Ramsden, 2003). Academic engagement relates to students’ behavior, cognitive activity, and emotions, all of which influence self-regulated learning. Students employing deep learning strategies demonstrate higher cognitive engagement, gaining deeper understanding by connecting knowledge points (Schaufeli et al., 2002; Fredricks et al., 2016). Academic engagement impacts learning approach, with actively engaged students displaying greater interest and self-regulation, particularly when motivated by the content (Bryson and Hand, 2007). Academic engagement correlates with self-regulation, as cognitive input shapes learning strategies, engagement, and willpower through self-monitoring and reflection, thereby influencing students’ learning approaches (Pintrich and De Groot, 1990; Li and Lajoie, 2021). Teachers can enhance academic engagement by employing effective strategies that foster positive learning experiences and promote beneficial learning approaches (Taghani and Razavi, 2021). While both teacher support and instructional strategies enhance academic engagement, they do so through different mechanisms. Teacher support addresses psychological needs and motivation, whereas instructional strategies focus on structuring curricula and fostering cognitive engagement through optimized relationships (Xu et al., 2024; Heilporn et al., 2021). Li and Lajoie (2021) demonstrated that academic engagement mediates the effect of Perceived teacher support on learning approach, but Ginting (2021) research highlights the complexity of this relationship, identifying academic engagement as a key determinant of academic achievement. Academic emotions have an impact on learning behaviors, and it has been found that high affective engagement reduces students’ academic shame and promotes active inquiry learning styles, and that negative emotions trigger avoidant learning strategies, such as procrastination (Chao et al., 2025). Student behavioral input affects students’ strategy choices, and studies have found that online whiteboards can optimize cognitive strategies for learning, such as the use of dialectical argumentation in learning, by facilitating interactive behaviors that push students to shift from passive acceptance to active construction. It was also found that students’ cognitive engagement also affects their metacognitive regulation, and that students with a high level of learning engagement are more adept at adapting their cognitive strategies through social interactions, leading to the formation of dynamic learning networks (Zheng et al., 2023) student emotional engagement also affects student learning persistence, and research has found that students who can shift negative emotions, such as academic shame, through self-regulation can reduce academic procrastination and maintain deep academic engagement (Yang et al., 2021). Motivational factors, self-regulation, and interest further mediate the relationship between academic engagement, perceived teacher support, and learning approach, suggesting the potential influence of other unexplored moderating variables.

### 1.3 The mediating role of achievement goal orientation

Achievement goal orientation refers to students’ understanding of the purpose and meaning of tasks and plays a crucial role in self-regulated learning (Pintrich, 2000). Achievement goal orientation consists of two primary goals: task-oriented goals and performance-oriented goals. In terms of motivation, achievement goal orientation includes mastery orientations, which focus on internal motivations related to learning activities, and performance orientations, which emphasize external motivations, such as how others perceive one’s performance (Elliot, 1999). Research indicates that task-approach goals positively guide learning, whereas avoidance goals negatively affect learning outcomes (Mädamürk et al., 2021; Miller et al., 2021). However, findings regarding mastery-avoidance goals and performance-approach goals are mixed. Some studies report no impact of mastery-avoidance goals on learning (Babenko et al., 2018), while others suggest positive (Pascarella, 2006) or negative effects (Lee and Anderman, 2020). Similarly, Performance-approach goals are associated with positive predictions of learning activities (Senko et al., 2013) and are linked to surface learning (Greene and Miller, 1996), though their relationship with students’ learning strengths and weaknesses remains inconsistent (Miller et al., 2021). Given these contradictions, this study examines both mastery-avoidance goals and performance-approach goals to clarify their impact on learning approaches in the context of perceived teacher support. Goal orientation can influence learning strategies by regulating the allocation of cognitive resources. Mastery-avoidance goals, in which cognitive resources are focused on “avoiding mistakes” rather than” exploring,” result in behaviors that inhibit students’ creative thinking and deep-processing strategies and lead to the emergence of surface learning strategies. The performance-approach goal is represented by cognitive resources focused on “optimizing results,” which corresponds to the behavioral outcome of preferring efficient strategies and neglecting deeper integration of knowledge (Ariani, 2023). Achievement goal orientation influences students’ learning strategies by enhancing metacognitive strategies through both mastery-avoidance goals and performance-approach goals. Additionally, perceived teacher support positively predicts mastery-avoidance goals and performance-approach goals, which, in turn, support metacognitive learning (Peng et al., 2022). Achievement goal orientations also moderate the effects of perceived teacher support on academic performance. Mastery-oriented students benefit more from teacher support, while performance-approach goals reduce the effectiveness of such support (Ouyang, 2005). Furthermore, Urdan and Kaplan (2020) highlighted that the impact of achievement goal orientation varies across racial, cultural, and gender groups, indicating a more complex mediating relationship. Interestingly, their findings contradict Pascarella (2006) conclusions, suggesting that additional, unexplored variables may influence the relationship between teacher support and students’ learning approaches.

### 1.4 Chain mediator of academic engagement and achievement goal orientation

A student’s academic engagement profile can influence their achievement goal orientation type, though few studies have explored this relationship. Some research highlights how adaptive behaviors predict motivational profiles. For example, a study on e-learning found that students’ academic engagement, including distraction levels, was a key factor affecting motivation (Dubey et al., 2019). Another study found that adaptive behaviors predicted achievement goal orientation, with task-based orientations being positively associated with devotional situations (Usán Supervía and Salavera Bordás, 2020). This suggests that academic engagement and achievement goal orientation could function as chain intermediaries. Recent findings show that digital academic engagement impacts learning differently based on motivational types. Performance-approach goal students prefer digital assignments and tools for superficial learning tasks, while mastery-oriented students use digital tools for self-improvement and restructuring cognitive styles (Mädamürk et al., 2021). In contrast, mastery-avoidance goal and avoidant students show less interest in digital tasks, likely due to contentment with their current learning approach or lack of interest in the activity itself (Mädamürk et al., 2021). Thus, academic engagement and achievement goal orientation (mastery-avoidance goals and performance-approach goals) likely play a chain-mediating role in the effect of PTS on LA among university students. Teachers support the enhancement of students’ sense of control over their learning and their intrinsic value identity by meeting their basic psychological needs. When students’ psychological needs are met, they are more inclined to adopt adaptive goals and improve their learning styles through self-regulation strategies (Bai and Gu, 2024).

According to Personal Investment Theory, meaning construction in learning arises from student-teacher interactions, which shape learning style choices. Students with higher academic scores perceive greater emotional teacher support, strengthening teacher-student relationships and motivation to learn (Lv and Liang, 2023). Learning style selection is influenced by subjective goals, reflected in achievement goal orientation, and students’ self-awareness, expressed through academic engagement. Goal orientations mediate the influence of teacher support on performance, revealing the motivational processes underlying perceived teacher support and learning approach (Yang et al., 2023). Emotional engagement also plays a role, mediating the impact of teacher support on learning (Ekatushabe et al., 2021). Motivation and engagement function as chain mediators, linking teacher support with student outcomes (Huang and Wang, 2023). Further research is needed to explore the chain mediation of academic engagement and achievement goal orientation in the relationship between perceived teacher support and learning approach, as gaps remain in the literature.

### 1.5 The present study

**Figure 1:**
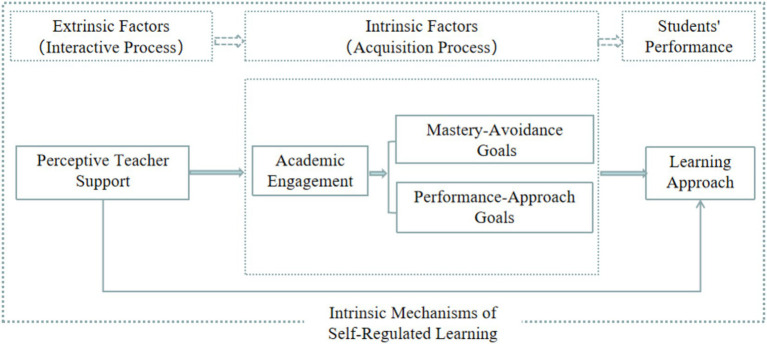
Conceptual analysis framework diagram.

This study tests four hypotheses: H1. University students’ perceived teacher support predicts their learning approach; H2. Perceived teacher support indirectly predicts learning approach through academic engagement; H3. Perceived teacher support indirectly predicts learning approach through mastery-avoidance goals and performance-approach goals; and H4. PTS indirectly predicts learning approach through the chain mediating effect of academic engagement and achievement goal orientation (mastery-avoidance goals and performance-approach goals). Figure 1 presents the hypothetical model, outlining how self-regulated learning activities connect perceived teacher support with learning approach through five variables across three systems: extrinsic factors, intrinsic factors, and student performance. Perceived teacher support reflects extrinsic factors, while academic engagement and achievement goal orientation (mastery-avoidance goals and performance-approach goals) represent intrinsic factors, with students’ learning approach manifesting in academic performance. The extrinsic factor captures the interaction between students and their environment, while the intrinsic factor reflects the cognitive process of forming students’ mental structures. Given the cognitive and motivational differences between deep and surface learning approaches, the model separates these constructs. Deep learning, driven by intrinsic motivation to deeply understand and integrate knowledge, contrasts with surface learning, motivated extrinsically and focused on task completion. This distinction allows for examining how perceived teacher support influences these learning approaches differently. The analysis measures the specific impact of perceived teacher support on each learning style, providing nuanced insights into how teachers can support different types of student engagement effectively.

## 2 Materials and methods

### 2.1 Participants

The study employed convenience sampling, targeting students majoring in literature and science across freshman, sophomore, and junior years at a university in Yuxi, Yunnan Province, China. This method was chosen due to the accessibility and willingness of the participants, which allowed for efficient data collection within the constraints of the study. A total of 558 questionnaires were distributed, with 543 valid responses returned. The questionnaire administration was conducted by the research team. Among the valid questionnaires returned, 192 (35.4%) were from first year students, 186 (34.3%) were from second year students and 165 (30.4%) were from third year students; 212 (39.0%) were men and 331 (61.0%) were women. From the valid questionnaires returned, it was found that the age distribution of students in the 3 years ranged from 18 to 24 years (*M* = 20.15 years, *SD* = 1.27 years). Descriptive statistics of the participants’ demographic variables are shown in Table 1.

**Table 1:** Demographic characteristics of the participants (*n* = 543).

Variables	Groups	Frequency (%)
Gender	Men	212 (39.0%)
	Women	331 (61.0%)
Grade level	First Year University Students	192 (35.4%)
	Second Year University Students	186 (34.2%)
	Third Year University Students	165 (30.4%)
Type of major	Liberal Arts Major	284 (52.3%)
	Science Major	259 (47.7%)

### 2.2 Measures

#### 2.2.1 Perceptions of teacher support scale

A Perceptions of Teacher Support scale developed by Babad (1990) and translated and revised by Chinese scholars was used (Sezgin Selçuk et al., 2009). The scale consists of 19 questions, divided into learning support (e.g., “*When I answer a question, right or wrong, my teacher will tell me*”), emotional support (e.g., “*I often feel that my teachers have high expectations of me*”), ability support (e.g., “*My teachers have always supported me in various activities and competitions*”). A 6-point scale is used to indicate the change in level from 1 (*not at all fit my situation*) to 6 (*completely fit my situation*), with higher scores indicating more positive perceptions of PTS. A test of the scale used in this research showed Cronbach’s *α* = 0.92. The reliability and validity of the scale has been confirmed in existing literature (Jia et al., 2020).

#### 2.2.2 Study engagement scale

Achievement engagement was assessed using a scale developed by Schaufeli et al. (2002). There were 17 questions on this scale covering vitality (e.g., “*Even when my studies did not go well, I was not discouraged and was able to persevere*”), dedication (e.g., “*I find the learning purposeful and rewarding*”), and absorption (e.g., “*I can hardly put down my studies*”). A 7-point scale is used, ranging from 1 (*never*) to 7 (*always*), with higher scores indicative of better academic engagement conditions. The reliability of the scale was strong, Cronbach’s *α* = 0.95. The scale has also been shown to have good reliability and validity in prior research (Pap et al., 2021).

#### 2.2.3 Achievement goal questionnaire

The motivation type was measured using the Achievement Goal Questionnaire, which was translated and adapted by Chinese scholars (Liu-Hui and Guo-De, 2003), based on the four-dimensional model of the achievement goal orientation constructed by Elliot and McGregor (2001). The questionnaire has 29 items, consisting of statements for mastery-avoidance goals (e.g., “*In exams, I often worry that I have misunderstood the requirements of the questions*”) and performance-approach goals (e.g., “*I always want to prove through my actions that I can do it*”). The questionnaire is scored on a 5-point, ranging from 1 (*very not fit for me*) to 5 (*very fit for me*). The higher the total score for a specific type of goal indicates that the subject belongs to that type of achievement goal A test of the scale’s reliability was conducted, finding a strong reliability: Cronbach’s *α* = 0.92. The scale has been shown to have good reliability and validity in prior research (Bai et al., 2013).

#### 2.2.4 Learning process questionnaire

This Learning Process Questionnaire developed by Kember et al. (2004) was used and consisted of 22 items divided into four learning types: deep motive (e.g., “*I often find that learning makes me feel happy and satisfied*”), deep strategy (e.g., “*I try to connect what I’ve learned in different courses*”), surface motive (e.g., “*I am discouraged by an unsatisfactory test score, and I worry about the next exam*”), surface strategy (e.g., “*I do not think there’s any point in studying for things that aren’t often tested on exams*”). The questionnaire is scored on a 5-point scale, from 1 (*never applicable*) to 5 (*always applicable*). A high total score on one dimension meant that the student was influenced most by that motivation to adopt the appropriate learning approach. Reliability for this measure was also strong: Cronbach’s *α* = 0.90. The scale has been shown to have good reliability and validity (Yin et al., 2015).

### 2.3 Procedure

The questionnaires were distributed on site with the consent of the study participants themselves. The researcher and a doctoral student in psychology, who was familiar with the content and structure of the questionnaire, as well as an instructor teaching a psychology-related course, were the main testers. Students responded anonymously and individually, and were allowed to stop answering the questionnaire at any time. At the end, all questionnaires were collected on the spot by members of the research team. The questionnaires were then checked and 15 invalid questionnaires were removed, resulting in 543 valid questionnaires, a 97.31% validity rate. Finally, the data were analyzed using SPSS 26.0, and the research model was tested through the SPSS macro program PROCESS (Hayes, 2012).

### 2.4 Statistical analysis

First, before formally starting the data analysis, the issue of common method bias of the variables was tested by Harman one-way method. If the number of selected factors is greater than 1 when exploratory factor analysis is used, and at the same time the variance explained by the first factor is less than the critical value, it means that the scientific validity and credibility of this study can be guaranteed. This means that there is no methodological bias problem in the data of this study. Secondly, correlation analyses were conducted on the relevant variables in the valid questionnaires and the mean values of the variables were calculated. Thirdly, the model was tested. The model was regressed using the SPSS macro program PROCESS. This study tests the chaining of two mediating variables, so PROCESS Model 6 was used. Model 6 was chosen to present the chain-mediating role of the two mediating variables, learning engagement and achievement goal orientation, in perceiving the effect of teacher support on learning styles. However, since two types of achievement goal orientations— mastery-avoidance goals and performance-approach goals—were chosen for this study, two chain-mediating paths would be presented. The regression test results were analyzed to see whether the model was validated.

### 2.5 Ethical approval

This investigation is concerned with human beings and therefore the study strictly follows the relevant requirements of the Declaration of Helsinki. The investigation was obtained permission from the Ethics Committee of the lead author (ERB No.2022035, dated: 13/10/2022). Prior to the administration of the questionnaire, the researcher informed the university students about the purpose of the questionnaire, the time needed for the questionnaire, and the use of the data collected by the questionnaire. To further enable students’ understanding of the intent of this study, the researcher offered university students an informed consent form and the study commenced upon the signature of it by the participants.

## 3 Results

### 3.1 Common method bias

In this investigation, data were collected using student self-assessment, which may result in common method bias. Therefore, common method bias was examined by Harman’s one-way. There were 12 eigenvalues greater than 1 found to explain 59.72% of the variance; and the first elements explained 21.33% of the variance, which was less than the critical value of 40%, demonstrating that the effect due to common method bias was not statistically significant. However, given that the data relied exclusively on self-report, the study further reduced the potential impact of common method bias through procedural controls. In terms of questionnaire design and optimization, the questionnaire was measured in stages, with the independent and dependent variables dispersed to different parts of the questionnaire to avoid logical correlation implication; the questionnaire design also focused on the semantic clarity of questionnaire entries, and pre-tested revisions of ambiguous expressions were made to ensure that entries were understood consistently and to reduce subjective interpretation bias among the subjects. In this study, anonymity and confidentiality were emphasized to the subjects in the process of data collection, and the subjects were clearly informed that the data were only used for academic research and anonymity was adopted, which would also reduce social desirability bias (Tourangeau and Yan, 2007).

### 3.2 Descriptive statistics analysis

Table 2 displays the correlation coefficients between the five measured variables. In terms of statistical significance, there were strong and significant correlations between all five variables (*p* < 0.01). Kim (2013) suggests that the data are non-normal when the skewness value is greater than or equal to 2 and the kurtosis value is greater than or equal to 7. In this study, the data skewness of the variables ranged from −0.56 to −0.05, while the kurtosis ranged from 0.07 to 1.13, all of which were below the recommended critical values for evaluating normality.

**Table 2:** Descriptive analysis of relevant variables.

Variables	*M*	*SD*	Skewness	Kurtosis	1	2	3	4	5
1. PTS	3.99	0.73	−0.56	1.13	1				
2. AE	4.16	0.97	−0.05	0.50	0.569**	1			
3. MAGs	3.48	0.65	−0.53	1.11	0.420**	0.429**	1		
4. PAGs	3.42	0.65	−0.38	0.80	0.499**	0.499**	0.642**	1	
5. LA	3.32	0.56	−0.05	0.07	0.541**	0.603**	0.552**	0.655**	1

M, mean; SD, standard deviation; PTS, perceived teacher support; AE, academic engagement; MAGs, mastery avoidance goals; PAG, performance approach goals; LA, learning approach. **p < 0.01.

### 3.3 The mediation role of academic engagement and achievement goal orientation

All analyses used the deviation-corrected nonparametric percentage Bootstrap routine to detect the intermediary in effect (see Table 3). Perceived teacher support, academic engagement, mastery-avoidance goals and performance-approach goals all independently predicted learning approach in university students. Model 1 showed that perceived teacher support positively predicted learning approach (*β* = 0.21, *p* < 0.001); perceived teacher support positively predicted academic engagement (*β* = 0.57, *p* < 0.001), perceived teacher support positively predicted mastery-avoidance goals (*β* = 0.26, *p* < 0.001); academic engagement positively predicted learning approach (*β* = 0.35, *p* < 0.001), mastery avoidance goals positively predicted learning approach (*β* = 0.31, *p* < 0.001); and academic engagement positively predicted mastery avoidance goals (*β* = 0.28, *p* < 0.001). From Model 2, it can be found that perceived teacher support positively predicts learning approach (*β* = 0.16, *p* < 0.001); perceived teacher support positively predicts academic engagement (*β* = 0.57, *p* < 0.001), perceived teacher support positively predicts performance approach goals (*β* = 0.32, *p* < 0.001); academic engagement positively predicts learning approach (*β* = 0.30, *p* < 0.001), performance approach goals positively predicts learning approach (*β* = 0.43, *p* < 0.001); and academic engagement positively predicted performance approach goals (*β* = 0.32, *p* < 0.001).

**Table 3:** Intermediary effects model testing.

Variable	AE				MAGs/PAGs				LA			
	*β*	*SE*	*t*	*R^2^*	*β*	*SE*	*t*	*R*2	*β*	*SE*	*t*	*R*2
Model 1				0.32				0.23				0.50
PTS	0.57	0.05	16.10***		0.26	0.04	5.67***		0.21	0.03	5.52***	
AE					0.28	0.03	6.12***		0.35	0.02	9.08***	
MAGs									0.31	0.03	9.03***	
Model 2				0.32				0.32				0.55
PTS	0.57	0.05	16.10***		0.32	0.04	7.36***		0.16	0.03	4.25***	
AE					0.32	0.03	7.34***		0.30	0.02	8.14***	
PAGs									0.43	0.03	12.14***	

PTS, perceived teacher support; AE, academic engagement; MAGs, mastery avoidance goals; PAGs, performance approach goals; LA, learning approach. ***p < 0.001.

**Figure 2:**
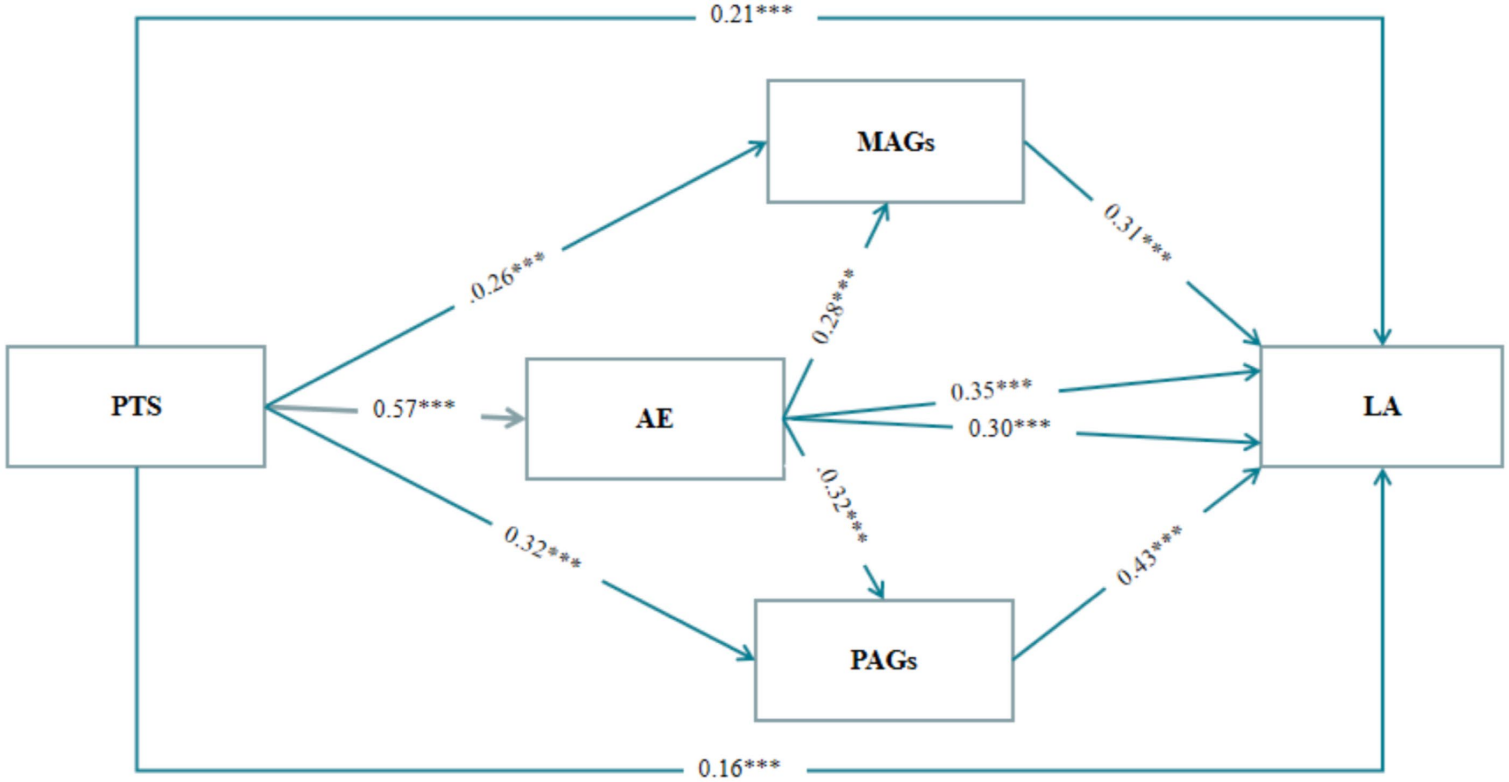
Diagram of the Model’s Path Coefficients with Mastery Avoidance Goals and Performance Approach Goals. PTS, perceived teacher support; AE, academic engagement; MAGs, mastery avoidance goals; PAGs, performance approach goals; LA, learning approach ***p < 0.001.

Thus, there is a chain mediating effect of academic engagements, mastery avoidance goals and performance approach goals in the effect of PTS on LA (see Figure 2).

In order to obtain credible results for mediating effects, the deviation-corrected nonparametric percentage Bootstrap procedure was further used to test intermediary effects, with 95% confidence intervals not including 0 indicating significant mediating effects. When the mediating effects of academic engagements and mastery avoidance goals were examined, a direct effect value of 0.16 was found for perceived teacher support on learning approach, accounting for 39.02% of the overall effect value, with a 95% Bootstrap confidence interval of [0.10, 0.22], indicating that the direct effect was significant. Each unit improvement in perceived teacher support directly drives 0.16 units of improvement in learning approach. There is a significant direct effect of perceived teacher support on learning approach, and even without accounting for the mediating variables, perceived teacher support still explains close to 40% of the effect. This means that direct interventions on the part of the teacher can significantly optimize the improvement in learning styles of about 16% of the students, even without taking into account the changes in their psychological state. The indicative effect value of the indirect effect of academic engagements and mastery avoidance goals in the effect of perceived teacher support on learning approach was 0.25, which accounted for 60.98% with a 95% Bootstrap confidence interval of [0.20, 0.30], indicating that the indirect effect was significant. This mediating event is composed of three pathways of independent effects: the respective values of the effects are 0.15, 0.06, and 0.04. The effect value of perceived teacher support influencing learning approach through academic engagements was 0.15, the effect value of perceived teacher support influencing learning approach through mastery avoidance goals was 0.06, and the chain mediation formed by academic engagements and mastery avoidance goals produced a chain mediation effect of 0.04 in perceived teacher support influencing learning approach (see Table 4). The fact that more than 60% of the effects were explained through mediation suggests that the perceived teacher support plays a more critical role in shaping students’ psychological mechanisms, with a particular focus on the two mediating mechanisms of academic engagements and mastery avoidance goals. Specifically, academic engagements are the strongest mediating pathway, with each 1-unit increase in perceived teacher support indirectly leading to a 15% increase in learning approach through academic engagements. This implies that teachers’ efforts to build trusting relationships and design interesting tasks to motivate students are close to the direct effect on optimizing students’ learning styles, i.e., the indirect effect is close to the direct effect. The presence of mastery avoidance goals, although small in magnitude, suggests that perceived teacher support reduces students’ avoidance tendency of“fear of failing, contributing to a 6% improvement. The chain of mediating roles of academic engagements and mastery avoidance goals is a small but important one, showing that teacher support can both directly improve input and directly improve learning through academic engagements. The small but significant chain mediating effect of academic engagements and mastery avoidance goals suggests that teacher support both directly enhances engagement and further reduces avoidance through engagement enhancement, creating a virtuous circle, implying that sustained teacher support can have long-term effects.

**Table 4:** Bootstrap analysis of significance tests for intermediate effects.

Path	Effect	Effect size (%)	Boot LLCI	Boot ULCI
Model 1				
PTS → AE → LA	0.15	36.59%	0.11	0.19
PTS → MAGs→LA	0.06	14.63%	0.04	0.09
PTS → AE → MAGs→LA	0.04	9.76%	0.02	0.06
Indirect effect	0.25	60.98%	0.20	0.30
Direct effect (PTS → LA)	0.16	39.02%	0.10	0.22
Total effect	0.41	100%	0.36	0.46
Model 2				
PTS → AE → LA	0.13	31.71%	0.09	0.17
PTS → PAGs→ LA	0.10	24.39%	0.07	0.14
PTS → AE → PAGs→ LA	0.06	14.63%	0.03	0.08
Indirect effect	0.29	70.73%	0.24	0.35
Direct effect (PTS → LA)	0.12	29.27%	0.06	0.17
Total effect	0.41	100%	0.36	0.46

PTS, perceived teacher support; AE, academic engagement; MAGs, mastery avoidance goals; PAG, performance approach goals; LA, learning approach; LL, lower level; CI, confidence interval; UL, upper level.

In addition, when testing the mediating effects of academic engagements and performance-approach goals, a direct effect value of 0.12 was found for perceived teacher support on learning approach, accounting for 29.27% of the overall affective value, with a 95% Bootstrap confidence interval of [0.06, 0.17], indicating that the direct effect was significant. For every 1 unit increase in perceived teacher support, the quality of learning styles directly increases by 0.12 units. The effect is small but significant, there is a significant direct effect of perceived teacher support on learning approach, and even without considering the mediating variables, perceived teacher support still explains close to 30% of the effect. This means that direct interventions on the part of the teacher can significantly optimize the improvement in learning styles of about 12% of the students, even without taking into account the self-regulation of the students. The indicative effect value of the indirect effect of academic engagements and performance-approach goals in the perceived teacher support on learning approach relationship was 0.29, which accounted for 70.73% with a 95% Bootstrap confidence interval of [0.24, 0.35], demonstrating the indirect effect was significant. This mediating relationship is composed of three pathways of independent effects: the respective values of the effects are: 0.13, 0.10, and 0.06. Of all the indirect effects, perceived teacher support had an effect value of 0.13 on learning approach through academic engagement, perceived teacher support had an effect value of 0.10 on learning approach through performance-approach goals, and perceived teacher support had an effect of 0.06 on learning approach through the chain mediation formed by academic engagement and performance-approach goals. The fact that 70% of the impact was explained through mediation suggests that student self-regulation has leverage, with a particular focus on two mediating mechanisms, academic engagements and performance-approach goals. Specifically, academic engagements are the strongest mediating pathway, with each 1-unit increase in perceived teacher support indirectly leading to a 13% increase in learning approach through academic engagements. This implies that the teacher’s indirect effect—optimizing students’ learning styles by motivating them to engage in learning—exceeds the direct effect. The presence of performance-approach goals suggests that perceived teacher support leads to the satisfaction of students’ sense of competence through the target pathway, contributing to a 10% improvement in learning styles. The chained mediation of the academic engagements and the performance-approach goals is small but important, showing that teacher support both directly enhances engagement, and, through engagement enhancement to further satisfy students’ sense of competence, forming a virtuous cycle, which implies that continuous teacher support can help students construct an autonomous growth ecosystem, and ultimately realize the transformation of teacher support into lasting learning enhancement through psychological mechanisms.

## 4 Discussion

Learning approach is a reflection of students’ learning strategies and is an important variable that influences their academic experience (Taghani and Razavi, 2021). Based on the Personal Investment Theory, students consider perceived teacher support on their learning approach. There are individual differences in the investment process for learning between students, mainly because individual investment decisions are influenced by three investment factors: perceived teacher support as a factor of perceived options; academic engagement as a factor of sense of self-beliefs; and achievement goal orientation as a factor of personal incentives.

### 4.1 The impact of perceived teacher support on learning approach

Teachers are the most helpful source in students’ learning experiences, and the findings of this study corroborate this. Students’ perceived teacher support positively predicted their learning approach, which is consistent with existing research findings (van Beek, 2015). Research has found that teachers’ use of process-oriented instruction promotes students’ monitoring and regulation of their learning activities, which helps students use appropriate thinking strategies to enhance their ability to construct and apply their knowledge (van Beek, 2015). Based on Personal Investment Theory, the possible reason for this outcome is that the interaction between students and teachers generates meaningful interpretations of learning content and learning approach, influencing students’ outward learning behavioral performance. Perceived teacher support is a perception made in a particular learning activity, where students perceive the support given by teachers as available and reasonable. Students, who like the support given by teachers, will then see the support given by teachers as a resource that they have. Students then invest in the teacher as a resource to influence their own learning activities, particularly their learning approach. Ultimately, this helps students interpret what support teachers can give to their learning activities and thus develop the ability to regulate their personal investment, which influences the learning approach they adopt. The impact of perceived teacher support on college students’ learning styles has expanded the research on the relationship between teachers’ teaching and students’ learning in the field of higher education, where previous studies focused more on how teachers perform, ignoring how college students may play an individualized role (Klem and Connell, 2004). This study emphasizes the importance of personalized support and pays more attention to the diverse learning styles and needs of university students.

The results indicate that perceived teacher support —both emotional and instructional—play a significant role in shaping learning approach. Specifically, students who perceived higher levels of teacher support were more inclined to adopt deep learning approaches. This was characterized by their willingness to engage critically with course material and a preference for understanding concepts thoroughly rather than merely memorizing facts. Additionally, the data suggest that students from backgrounds with traditionally lower access to educational resources particularly valued emotional support from teachers, which significantly correlated with their preference for deep learning strategies. This aligns with Personal Investment Theory, which posits that individual perception of support and resources can greatly influence one’s engagement and investment in learning tasks (Zhao and Liu, 2022). Furthermore, our analysis showed that while most participants demonstrated a propensity for deep learning under supportive conditions, there were variations based on year of study and major. For instance, seniors and students in humanities were more likely to engage in deep learning compared to freshmen and those in more technical fields, suggesting that maturity and field of study may interact with perceived teacher support to influence learning approaches. These findings underscore the complexity of learning approaches among university students and highlight the critical role of teacher support in fostering an environment conducive to deep learning. They not only reinforce the theoretical assertions of Personal Investment Theory but also offer practical insights for educators looking to enhance instructional strategies and support mechanisms to cater to diverse learning needs.

### 4.2 Chain mediating role of academic engagement and achievement goal orientation

Academic engagement moderates the effect of perceived teacher support on university students’ learning approach; and university students’ perceived teacher support has an indirect effect on their learning approach through academic engagement. This conclusion has been supported by existing studies (Taghani and Razavi, 2021). It was found that academic engagement positively predicted students’ learning approach, and that students were more susceptible to utilizing deeper learning styles and learn by focusing on the connections between old and new knowledge if they were more cognitively engaged in the learning activities (Fredricks et al., 2004). Students’ academic engagements are malleable; teachers who take a process-oriented teaching style to student learning will likely promote metacognitive activities, involving higher level thinking activities among students (van Beek, 2015). This means self-beliefs enriched the student’s academic engagement and thus can put greater effort into their learning and use of a specific learning approach.

Perceived teacher support can also influence university students’ learning approach through achievement goal orientation, and this study found that both mastery-avoidance goals and performance-approach goals in the achievement goal orientation correctly predicted university students’ learning approach, a finding that echoes existing research (Pascarella, 2006; Senko et al., 2013). The present study also found that the predictive effect of performance-approach goals was greater than that of mastery-avoidance goals on learning approach in university students. This may be related to why performance-approach goals tend to trigger students’ surface learning approach (Greene and Miller, 1996). The mastery-avoidance goals and performance-approach goals, as mediating variables, modulate the effectiveness of perceived teacher support on learning approach in university students. This helps demonstrate the importance of the supportive role teachers can have at the individual student level. Moreover, studies have found that instructors’ teaching can stimulate the development of metacognitive learning strategies by influencing students’ achievement goal orientation (Ariani, 2023). Achievement goal orientation can be seen as the mental energy needed for learning. Meeting students’ needs—whether performance-oriented or mastery-oriented drive their pursuit to new knowledge or skills, maintaining the balance of cognitive energy is important for students’ learning. From the perspective of Personal Investment Theory, a possible reason for this difference in achievement goal orientation is that, as a personal incentive it helps students to identify the reasons for undertaking learning activities, which influences students’ interpretation of the effectiveness of teacher support. In this context, they may perceive the teacher as a facilitative resource and be encouraged to invest in the teachers’ directions as an influence on their learning approach.

Perceived teacher support can also influence students’ learning approach through the linking and intermediating processes of academic engagements and achievement goal orientations. It was found that perceived teacher support can positively predict students’ learning approach through the chain mediating effect of academic engagements and achievement goal orientations (mastery-avoidance goals and performance-approach goals). Although few studies have focused on this chain mediating effect, a finding that echoes existing research have found that individuals’ dedication behavior motivates individual mastery-oriented achievement goals (Usán Supervía and Salavera Bordás, 2020). This result, which can be explained by Personal Investment Theory, is that if the sense of self in a given environment is based on support and they believe that this support works, then the individual is likely to find the environment meaningful and invest more personal resources into it (Duda et al., 1989). A student’s perception of teacher support can be seen as an interaction between the student and the surroundings that allows academic engagement and achievement goal orientations to be activated in an integrative way. Specifically, students’ academic engagement if based on the support given by the teacher, and student recognition of the impact of the teacher’s contribution on their academic engagement and achievement goal orientations, then they will perceive learning activities with teacher support as meaningful and the student will be more willing to invest external and intrapersonal resources into the impact on their learning approach.

Previous studies have explored the effects of academic engagement (Li and Lajoie, 2021) and achievement goal orientation (Ariani, 2023) on LA respectively, but lacked a comprehensive model to explain the relationship between the two. The chain mediation between the two in this study constructs a more contextualized framework, which is helpful in systematically revealing the intrinsic mechanisms between academic engagement and achievement goal orientations, and highlights the complex interaction between the perceived teacher support and learning approach, as well as how student individualization influences play a role in this process. As a result, this study extended the existing literature on the influence mastery-avoidance goals and performance-approach goals have on students’ learning activities and strategies. The chain mediation by academic engagement and achievement goal orientation in the influence of perceived teacher support on learning approach provide an important basis and practical guidance for the development and implementation of perceived teacher support. By enhancing academic engagement and guiding positive achievement goal orientations, perceived teacher support can help students form an effective and learning approach improving their academic performance and learning ability. Future educational strategies should continue to explore the mechanisms of these factors to better serve educational practice.

## 5 Study limitations and future research implications

There are several limitations of this study to be acknowledged. First, this study included self-reporting data which might have potential response bias, social desirability bias. Second, the cross-sectional research design limits to interoperate the causal relationships among the study variables. Therefore, future longitudinal research should be considered to address this limitation. Third, the study recruited the participants from one university in Yuxi, China through convenience sampling technique which restricts the greater generalizability to broader context. Despite these limitations, our findings contribute to the broader discourse on educational psychology by highlighting the nuanced roles that perceived teacher support plays in shaping student learning approaches. By integrating academic engagement and achievement goal orientation as mediating factors, our study not only corroborates previous research suggesting the pivotal role of teacher support in educational settings but also offers new insights into the mechanisms through which this support influences students. The practical implications of these findings are significant. For educators, understanding the specific pathways through which support impacts learning can inform more effective pedagogical strategies that cater to diverse student needs. For policymakers, our results underscore the importance of fostering environments that facilitate comprehensive teacher support systems. The implications of our findings are multifaceted, extending beyond the academic community to influence educational practice and policy:

First, this study underscores the critical role of teacher support in fostering deep learning approaches among students. Educators should consider adopting more personalized support strategies that consider individual student needs, preferences, and background. Specifically, training programs for teachers could incorporate modules on emotional intelligence and communication skills to enhance their ability to provide effective support. Additionally, educational institutions should encourage a culture of support where teachers are recognized and rewarded for effectively engaging with students in a supportive manner. Second, at the policy level, our findings suggest the need for educational frameworks that prioritize and standardize teacher support as a key quality metric in teaching evaluations. Educational policymakers could consider developing guidelines that require institutions to implement support-centric teaching assessments. Moreover, funding allocations could be adjusted to support programs that train teachers in the identified effective support techniques, potentially enhancing the overall quality of education.

There are still aspects of future research that we need to continue to explore. The relationship between teacher support and student learning approaches invites further exploration across different educational levels and cultural contexts. Future research could investigate how these dynamics play out in primary and secondary educational settings, or in non-traditional learning environments such as online education platforms. Additionally, longitudinal studies could provide deeper insights into the long-term effects of perceived teacher support on student educational trajectories, offering a more comprehensive understanding of its impact. By acting on these implications, educational stakeholders can better align their strategies to foster environments that are conducive to effective learning, thereby enhancing educational outcomes and student satisfaction. Future trends of this study should incorporate multiple perspectives such as cognitive neuroscience, sociolinguistics and technology ethics, such as exploring how neuroplasticity theory can reshape differentiated instructional strategies, or analyzing the reconfiguration of classroom interaction paradigms by language practices of digital natives. More importantly for future research, mechanisms need to be established to translate these interdisciplinary concepts into modules for pre-service and post-service teacher professional development, and by designing practice platforms for teacher professional development, teachers can systematically integrate neuropedagogical findings, digital literacy frameworks, and culturally responsive pedagogies so as to develop more adaptive pedagogical intelligences in a complex educational ecology. The findings of this study prompt us to re-examine the pedagogical paradigm in teachers’ daily practice, and to question the traditional teaching paradigm centered on the transmission of knowledge when the synergistic effect of teachers’ affective support and students’ cognitive strategies has been empirically corroborated.

## 6 Conclusion

The conclusions have four facets: (1) Perceived teacher support was able to directly and positively predict learning approach in university students; (2) Perceived teacher support indirectly and positively predicted learning approach in university students through academic engagement; (3) Perceived teacher support can indirectly and positively predict learning approach in university students through mastery-avoidance goals and performance-approach goals; (4) Perceived teacher support can also indirectly and positively predict learning approach in university students through the linking intermediary function of academic engagements, mastery-avoidance goals, and performance-approach goals. Such results have helped bring clarity to the intrinsic mechanism by which perceived teacher support influences learning approach in university students. From this intrinsic mechanism, university students regulate their learning through academic engagement and achievement goal orientation monitoring. This study also gives some insights to university teachers, who should be more aware of students’ academic engagement in their learning process and work to guide them to establish positive achievement goals. This research also has important implications for universities’ efforts to improve teachers’ literacy in guiding students’ effective learning. This study endeavors to explore the detailed comprehension of effect on perceived teacher support on learning approach, and complementing the research on the relationship between teachers’ teaching and students’ learning in the higher educational settings. This study emphasizes the importance of personalized support and pays more attention to the diverse learning styles and needs of university students. This study found that university students personalized differentiated learning styles and needs, and that these differences reflected differences in students’ motivational and affective dimensions (i.e., the effects of the mediating role of the academic engagement and achievement goal orientation chains). The study of this mediating role helps to explore and clarify the mechanisms inherent in the complexity of teacher-student interactions (i.e., to clarify how these individual differences modulate the effects of perceived teacher support on learning approach). Overall, the findings of this study help to reveal and explain the intrinsic mechanisms of the complex interaction process of perceived teacher support affecting the learning approach of university students.

## Data Availability

The raw data supporting the conclusions of this article will be made available by the authors, without undue reservation.
